# Microbial Functional Genes Help Explain Soil Carbon Sensitivity to Temperature Across Global Biomes

**DOI:** 10.1111/gcb.71101

**Published:** 2026-09-23

**Authors:** Tadeo Sáez‐Sandino, Brajesh K. Singh, Chao Xiong, Juntao Wang, Chengjie Ren, Manuel Delgado‐Baquerizo

**Affiliations:** ^1^ Hawkesbury Institute for the Environment Western Sydney University Penrith New South Wales Australia; ^2^ School of Agriculture and Environment, Institute of Agriculture University of Western Australia Crawley Western Australia Australia; ^3^ State Key Laboratory for Crop Stress Resistance and High‐Efficiency Production, College of Agronomy Northwest A&F University Yangling Shaanxi China; ^4^ Laboratorio de Biodiversidad y Funcionamiento Ecosistémico Instituto de Recursos Naturales y Agrobiología de Sevilla (IRNAS), CSIC Sevilla Spain

**Keywords:** global mapping, metagenomic, microbial functional traits, microbial respiration, *Q*_10_ values, soil carbon sensitivity, temperature sensitivity

## Abstract

The temperature sensitivity of soil microbial respiration (*Q*_10_) is a major factor underpinning global soil carbon emissions under climate change. While microbial biomass is a key driver of *Q*_10_ values, the role of microbial functional traits in explaining *Q*_10_ values remains largely undetermined. Here, we combined temperature‐gradient incubations with a metagenomic database generated from a collection of surface soils collected across 196 natural ecosystems spanning all major biomes. We found that microbial functional traits related to carbohydrate metabolism and hemicellulose degradation, quantified as transcripts‐per‐million (TPM), are significantly correlated with *Q*_10_ values. Moreover, *Q*_10_ values display abrupt non‐linear shifts once thresholds of microbial functional traits are crossed. Global mapping further provides evidence that microbial functional traits associated with carbon degradation and high *Q*_10_ values are concentrated within the planet's largest soil carbon reservoirs at high latitudes in the Northern Hemisphere. These regions could represent critical hotspots of vulnerability to soil carbon losses under climate warming. Our results provide a new step towards incorporating metagenomic data and non‐linear microbial responses into next‐generation carbon–climate models.

## Graphical abstract

Temperature‐gradient incubations across 196 natural ecosystems combined with metagenomic data reveal microbial functional traits associated with soil *Q*_10_. These traits converge into complementary metabolic strategies involving carbon acquisition, carbon assimilation, and cellular maintenance and stress responses. The balance among these complementary strategies may contribute to variation in *Q*_10_ values.

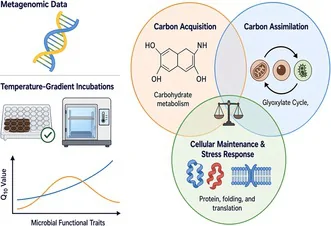

## 1 Introduction

Soils represent the largest active reservoir of carbon on Earth and underpin multiple functions essential for sustaining life on our planet, from climate regulation to soil fertility that sustains plant growth and ecosystem health (Kopittke et al. 2022). Carbon losses from soils to the atmosphere are primarily driven by the respiration of heterotrophic microbial communities, a flux so large that it releases approximately five times more CO_2_ annually than anthropogenic emissions (García‐Palacios et al. 2021). Current modelling and empirical evidence indicate that soil microbial heterotrophic respiration increases under warmer soil conditions, leading to enhanced carbon losses with important implications for climate–carbon feedback (Karhu et al. 2014). A major uncertainty in this context lies in predicting the temperature sensitivity of soil heterotrophic respiration, commonly expressed as the *Q*_10_ factor—the proportional increase in microbial respiration rates for every 10°C rise in temperature. Most recent studies suggest that microbial biomass and taxonomic composition associated with the soil microbiome are the most important microbial factors associated with *Q*_10_ values, along with substrate availability, mineral protection or environmental conditions (Haaf et al. 2021; Sáez‐Sandino et al. 2023). For instance, the mineral‐associated to particulate organic carbon (MAOC : POC) ratio provides a useful proxy for physicochemical mechanisms (e.g., occlusion in aggregates and formation of organo‐mineral associations) that constrain microbial access to carbon substrates (Dungait et al. 2012). However, much less is known about how microbial functional traits shape *Q*_10_ values across global scales. Unraveling the microbial functional traits behind *Q*_10_ responses is critical, as even small shifts in these values under rising global temperatures could determine whether terrestrial ecosystems act as net carbon sources or sinks (García‐Palacios et al. 2021).

Recent advances in soil metagenomics provide a powerful framework to disentangle the contribution of microbial functional traits to global *Q*_10_ patterns (Wang et al. 2021). A critical first step is to identify which microbial functional traits show positive or negative associations with *Q*_10_ patterns. For instance, soil microbes harbour a large array of genes for carbon degradation (e.g., xylA, encoding xylanase for hemicellulose breakdown) and assimilation (e.g., mcl, encoding malyl‐CoA lyase in the glyoxylate cycle), which may play pivotal roles in shaping *Q*_10_ values (Bankeeree et al. 2022; Zarzycki et al. 2007). Moreover, recent evidence indicates that the relationship between ecosystem respiration and warming is not always gradual, but can undergo abrupt shifts (i.e., temperature thresholds) (Mahecha et al. 2010). Such evidence implies that changes in microbial functional traits may lead to drastic non‐linear changes in *Q*_10_ values, which could lead to underestimation of CO_2_ emissions under warming scenarios. To the best of our knowledge, no study has attempted to establish relationships between microbial functional traits and *Q*_10_ values across contrasting regions, largely due to the high cost and effort required to generate high‐resolution functional genomic data. Local‐scale analyses cannot capture the abundance of different microbial functional traits across ecosystems and therefore provide limited insight into the global distribution of vulnerable regions. The absence of a global atlas identifying microbial functional traits and *Q*_10_ values constrains our ability to target the ecosystems most at risk of warming‐induced carbon losses (Jung et al. 2021), representing a major gap for conservation planning and climate policy. Given that non‐linear responses are rarely considered in current Earth system models, which still assume a constant *Q*_10_ value despite strong evidence of variability worldwide (Mahecha et al. 2010), integrating metagenomic insights into predictive frameworks is an urgent challenge for ecology science.

Here, we assess the contribution of microbial functional traits to the temperature sensitivity of soil respiration across contrasting terrestrial ecosystems. We operationally define microbial functional traits as the TPM‐normalized abundance of functional genes and metabolic pathways (Transcripts Per Million; TPM). These traits were characterized at multiple levels of biological organization, including KEGG Level 2 metabolic pathways (e.g., carbohydrate metabolism), ecologically meaningful gene sets (e.g., hemicellulose and starch degradation), individual functional genes (e.g., xylA, xylF and xylH involved in hemicellulose degradation) and carbohydrate‐active enzyme families (CAZy) (see Table S1 for a detailed lists of pathways, individual genes, and enzyme descriptions). Our metagenomic approach relies on microbial functional genes annotated in the Kyoto Encyclopedia of Genes and Genomes (KEGG), enabling a hierarchical characterization of microbial metabolism (Kanehisa and Goto 2000). To this end, we used two global databases available online comprising metagenomic data (Chen et al. 2026) and *Q*_10_ values (Sáez‐Sandino et al. 2023), respectively, and identified the overlapping soil samples for which both measurements were available. These matched samples represented a collection of soils from 196 natural ecosystems spanning all major climatic zones, vegetation types and contrasting soil properties (see Figure S1 for sampling locations). In brief, *Q*_10_ values were determined in a short‐term laboratory incubation (10 h) at four temperatures (0°C, 10°C, 20°C and 30°C) (Sáez‐Sandino et al. 2023). We aimed to (i) test which microbial functional traits are most strongly associated with *Q*_10_ values and whether these relationships are linear or non‐linear, and (ii) determine where high *Q*_10_ values spatially co‐occur with high TPM‐normalized abundances of microbial functional traits. We define thresholds as points in microbial functional traits at which *Q*_10_ values exhibit abrupt shifts (discontinuous thresholds or breakpoints) (Berdugo et al. 2020). If preserved across ecosystems, these thresholds may reveal inherent non‐linear dynamics often overlooked in ecological studies (Qi et al. 2002; Scheffer et al. 2009). Finally, because mineral protection is a key regulator of soil carbon persistence and *Q*_10_ values, we also examined which microbial functional traits are most strongly associated with the MAOC : POC ratio.

## 2 Material and Methods

### 2.1 Study Site and Soil Sampling

This study used a collection of 196 surface soils (collected up to 10 cm depth) originally collected during field surveys reported in Maestre et al. (2022), Delgado‐Baquerizo et al. (2021), Delgado‐Baquerizo et al. (2019) and Eldridge et al. (2023). This study includes overlapping samples between two global databases available online, for which metagenomic data were reported by Chen et al. (2026) and *Q*_10_ values were reported by Sáez‐Sandino et al. (2023). The MAOC : POC ratio was also available for a subset of 179 samples (Sáez‐Sandino, Gallardo, et al. 2024; Sáez‐Sandino, Maestre, et al. 2024), determined using size‐fractionation methods (Sokol et al. 2019). The sampling locations spanned a broad geographic range (64° N–62° S, 147° E–159° W) and encompassed contrasting climate zones (tropical, temperate, continental, polar and arid) and major vegetation types (forests, grasslands, shrublands and moss heath). For each location, geographic coordinates were recorded using a portable GPS. We focused on surface soils because topsoils represent the most biologically active layer, with dense root systems, high microbial biomass, labile nutrient pools and strong interactions with the atmosphere (Sáez‐Sandino, Gallardo, et al. 2024; Sáez‐Sandino, Maestre, et al. 2024). After collection, soils were divided into two subsamples: for subsequent analyses: one portion was air‐dried for the determination of soil physical and chemical properties, while the other was stored at −20°C for molecular analyses. This dual‐preservation approach is standard in global surveys and enables robust comparisons across sites. Samples were imported to Spain using letters of authority from the *Ministerio de Agricultura, Pesca y Alimentación*.

### 2.2 Metagenomic Sequencing and Bioinformatic Analyses

Soil DNA was extracted from ~0.5 g of frozen soil using the PowerSoil DNA Isolation Kit (Mo Bio Laboratories, Carlsbad, CA, USA), following the manufacturer's protocol, but using PCR water in the last step of this protocol. Metagenomic sequencing was carried out at the Next Generation Sequencing Facility (Western Sydney University, Australia) on an Illumina NovaSeq platform, generating ~10 Gb of high‐quality 150 bp paired‐end reads per sample. Raw reads were quality‐filtered with fastp (v0.20.1) to remove adapters and low‐quality sequences, and the resulting clean reads were assembled with MEGAHIT (v1.2.9) (Li et al. 2015), retaining only contigs ≥ 1000 bp. Open reading frames (ORFs) were identified using Prodigal (v2.6.3), and clustered into a non‐redundant gene catalogue at 95% sequence identity using CD‐HIT (Fu et al. 2012). Gene abundance was estimated as read counts and normalized to reads per million (RPM) with Salmon (v1.10.3) (Patro et al. 2017). Functional annotation of the non‐redundant gene set was performed against the eggNOG database (v5.0) using eggnog‐mapper (v2.1.12) (Huerta‐Cepas et al. 2019), retrieving KEGG Orthology (KO), KEGG pathway and CAZy family profiles (Kanehisa et al. 2017). TPM‐normalized abundance was used to normalize microbial functional gene abundances across metagenomic samples. TPM accounts for gene length and sequencing depth, enabling quantitative comparisons of normalized gene abundance among samples. To focus on microbial functions relevant to soil biogeochemistry, genes associated with human disease or plant/macroorganism systems were excluded from further analyses. Similarly, we also compiled a complete list of individual CAZy enzymes used in our analyses, along with their associated functional descriptions. Following DNA extraction in Spain, the samples were transferred to Australia for sequencing under the relevant permits issued by the Australian Department of Agriculture, Fisheries and Forestry. We also consulted the Nagoya Protocol focal points in each participating country to ensure that the sampling, transfer and subsequent use of genetic resources complied with applicable Nagoya Protocol requirements. Quantitative PCR (qPCR) data quantifying the absolute abundance of total bacteria were available for a subset of 173 samples (Xiong et al. 2026). Using the bacterial primers *Eub338/Eub518* (Fierer et al. 2005), reactions were prepared in a final volume of 10 μL containing 1 μL of DNA template, 5 μL of 2× PowerUp SYBR Green Master Mix (Thermo Fisher Scientific), 3.2 μL of PCR‐grade water and 0.4 μL of each primer. qPCR assays were performed on a LightCycler 480 system (Roche), and standard curves were generated using tenfold serial dilutions of plasmid DNA.

### 2.3 Experimental Incubations

Soil heterotrophic respiration data were obtained from Sáez‐Sandino et al. (2023), following a short‐term incubation (10 h) based on the MicroResp protocol (Campbell et al. 2003). This approach is a high‐throughput colorimetric method widely used for measuring soil respiration in microcosms (Han Weng et al. 2017). In this study, we used a total of 196 incubations performed at four target temperatures (0°C, 10°C, 20°C and 30°C), each with three replicates. To allow microbial reactivation after rewetting (Dacal et al. 2019), air‐dried soils were pre‐incubated for 5 h at 60% of water‐holding capacity, adjusted with sterile deionized water. Water‐holding capacity (WHC) was measured experimentally in the laboratory for each air‐dried soil using the funnel drainage method (Delgado‐Baquerizo et al. 2020). Following pre‐incubation, soils were incubated for 5 h with colorimetric detector plates containing cresol red, which changes colour as respired CO_2_ reacts with bicarbonate in the detection solution. Absorbance was recorded at 570 nm, and respiration rates (μg CO_2_‐C g^−1^ h^−1^) were calculated using a calibration curve (Campbell et al. 2003). Our pre‐incubation design minimized CO_2_ flushes associated with the Birch effect while maintaining microbial activity under controlled conditions (Fraser et al. 2016). Prolonged laboratory incubations may substantially deplete available carbon pools, thereby alter microbial activity and bias the results (Dacal et al. 2019). Consequently, temperature sensitivity of microbial respiration (*Q*_10_) was estimated from the exponential relationship between respiration rates (*R*_s_) and temperature (*t*):

$$R_{s}=R_{0}\times \exp\left(\beta \times t\right)\to Q_{10}=\exp\left(10\times \beta \right)$$

where *R*_s_ is the microbial respiration rate at temperature (*t*), *R*_0_ is the basal respiration, and *β* is the fitted temperature coefficient (i.e., the slope of the relationship between respiration and temperature on the exponential scale).

### 2.4 Statistical Analysis

#### 2.4.1 Random Forest

We employed random forest to identify the main microbial traits associated with *Q*_10_ values. Random forest generates an ensemble of classification trees using binary splits (Breiman 2001). Each tree is trained on a randomly selected subset of data (approximately two‐thirds), leaving the remaining one‐third as out‐of‐bag (OOB) samples for model validation. Predictor importance is quantified as the decrease in prediction accuracy (increase in mean squared error) when the data for a given variable is randomly permuted. The final importance score corresponds to the average decline across all trees. Analyses were implemented using the R package *spatialRF* (Benito 2021), which reduces multicollinearity, identifies relevant variable interactions and assesses model transferability through spatial cross‐validation.

#### 2.4.2 Global Mapping

Given its ability to capture non‐linear relationships, we also applied random forest regression to predict both the most important microbial traits and the spatial distribution of *Q*_10_ values. For this analysis, we included the 10 environmental factors, including climatic data (mean annual temperature [MAT], mean annual precipitation [MAP], mean diurnal range [MDR], temperature seasonality [TSEA], and precipitation seasonality [PSEA]), geographic factors (slope and elevation), and soil properties (soil pH, fine texture and soil carbon). Climatic data were obtained from the WorldClim database (Fick and Hijmans 2017), geographic information was retrieved from the Advanced Land Observation Satellite (Hamazaki 1999), and soil properties (soil pH, fine texture and soil carbon) were obtained from SoilGrids (Hengl et al. 2017). Variance inflation factors (VIFs) were calculated to assess potential collinearity among explanatory variables in our models. All variables exhibited VIF values below 5, indicating low correlation and minimal multicollinearity (Graham 2003).

The random forest model was constructed using 999 trees and 50 repetitions to maximize predictive performance on the training dataset. To identify regions where predictions could be less reliable, we calculated Mahalanobis distance in the multidimensional predictor space and excluded pixels exceeding the 99% *χ* ^2^ threshold from the final global predictions (shown in white in the maps). This approach restricts predictions to regions within the environmental space represented by the training data, thereby reducing the risk of extrapolation into environmentally novel conditions (Mallavan et al. 2010). Nevertheless, regions with limited spatial sampling may retain additional uncertainty even when their environmental conditions fall within the range represented by the training data. For *Q*_10_, the maximum variability among cross‐validation predictions was 0.125 (Figure S9), indicating low model‐to‐model variability. Model validation was performed by comparing predicted versus observed values following established procedures (Piñeiro et al. 2008). To further evaluate the predictive ability of our models, we conducted an independent random holdout validation. The resulting datasets were randomly divided into two subsets, with 70% of observations used for model training and the remaining 30% reserved for independent evaluation. Random Forest models were trained using the complete set of environmental predictors and subsequently used to generate predictions for the held‐out observations. Model performance was assessed using root mean squared error (RMSE), mean absolute error (MAE) and the coefficient of determination (*R* ^2^) calculated from the independent test data (Table S6).

We assessed spatial dependence in each response variable by calculating Moran's *I* across the sampling sites using *k*‐nearest‐neighbour networks with *k* set to 3, 5 and 8. Sampling coordinates were transformed to a projected coordinate system in metres before defining the spatial neighbourhoods, and the resulting spatial weights were standardized by row. Statistical significance was determined through permutation tests using the *spdep* package in R (Bivand and Wong 2018). Moran's *I* values greater than zero indicate positive spatial autocorrelation and clustering; values close to zero are consistent with a random spatial pattern, whereas negative values indicate spatial dispersion. To determine whether the environmental predictors included in the Random Forest models captured the main spatial structure, we also calculated Moran's *I* for the out‐of‐bag (OOB) residuals. A decrease in, or lack of, significant spatial autocorrelation in these residuals was taken as evidence that the predictor variables accounted for a substantial proportion of the underlying spatial structure (Table S6).

Additionally, we created three bivariate maps to spatially visualize relationships between microbial functional traits and *Q*_10_ values, respectively. These maps were generated using the *bivariate. map function*, which classifies each variable into deciles (10 quantiles) and assigns colours based on their joint quantile positions, providing an intuitive visualization of co‐distribution patterns across contrasting ecosystems (Guirado et al. 2022). To represent the uncertainty maps (Figures S10 and S11), we used the standard deviation of the model predictions obtained in the 5‐fold cross‐validation (Kohavi 1995). For each pixel, we calculated the mean predicted value and its associated standard deviation across the five predictive models. This per‐pixel standard deviation was interpreted as a spatial measure of model uncertainty, with higher values indicating lower prediction confidence (Guirado et al. 2022).

#### 2.4.3 Non‐Linear Regressions and Threshold Identification

To examine how microbial traits are associated with *Q*_10_ values, we followed a two‐step model‐selection framework based on Akaike's information criterion (AIC), adapted from (Berdugo et al. 2020). This approach first determines whether the relationship is better described as linear or non‐linear and then identifies the location and type of the threshold when evidence of non‐linearity is present. Before model fitting, bivariate outliers were identified and removed using Mahalanobis distance, with the 0.975 quantile of the corresponding *χ* ^2^ distribution used as the cutoff. This procedure was applied to reduce the influence of extreme observations on the estimated global relationships. Model performance was assessed using AIC scores within a 1000‐iteration bootstrap resampling procedure.

In the first step, we compared three alternative models: linear models (GLMs), quadratic polynomial regressions and generalized additive models (GAMs) fitted using the *mgcv* package (Wood 2025). A model was selected as the best fit for a given trait based on the highest frequency of selection across bootstrap iterations, considering models with ΔAIC < 2 as equivalent and selecting the more parsimonious model when applicable. In the second step, for relationships in which the GAM was selected as the best model, we evaluated whether the non‐linear pattern could be represented by an explicit threshold. We evaluated four alternative models using the *chngpt* package (Fong et al. 2017): *Segmented* models (continuous piecewise linear models capturing abrupt shifts in slope); *Step* models (discontinuous shifts representing sudden regime changes); *Stegmented* models (combining both discontinuity and slope changes), and *GAMs*. Model selection was again based on the lowest AIC across 1000 bootstrap iterations. The threshold model with the highest selection frequency was retained for subsequent threshold estimation. When GAM was retained as the best non‐linear model, a segmented model was used to obtain an explicit threshold location, representing the point of maximum change in the fitted relationship. Threshold locations were then estimated using 200 bootstrap iterations, from which standard deviations and 95% confidence intervals were calculated. Finally, to assess whether the identified thresholds corresponded to changes in the relationship between microbial traits and *Q*_10_ values, we separately fitted linear models to observations below and above each threshold and evaluated the resulting slope distributions using bootstrap resampling.

## 3 Results and Discussion

**FIGURE 1:**
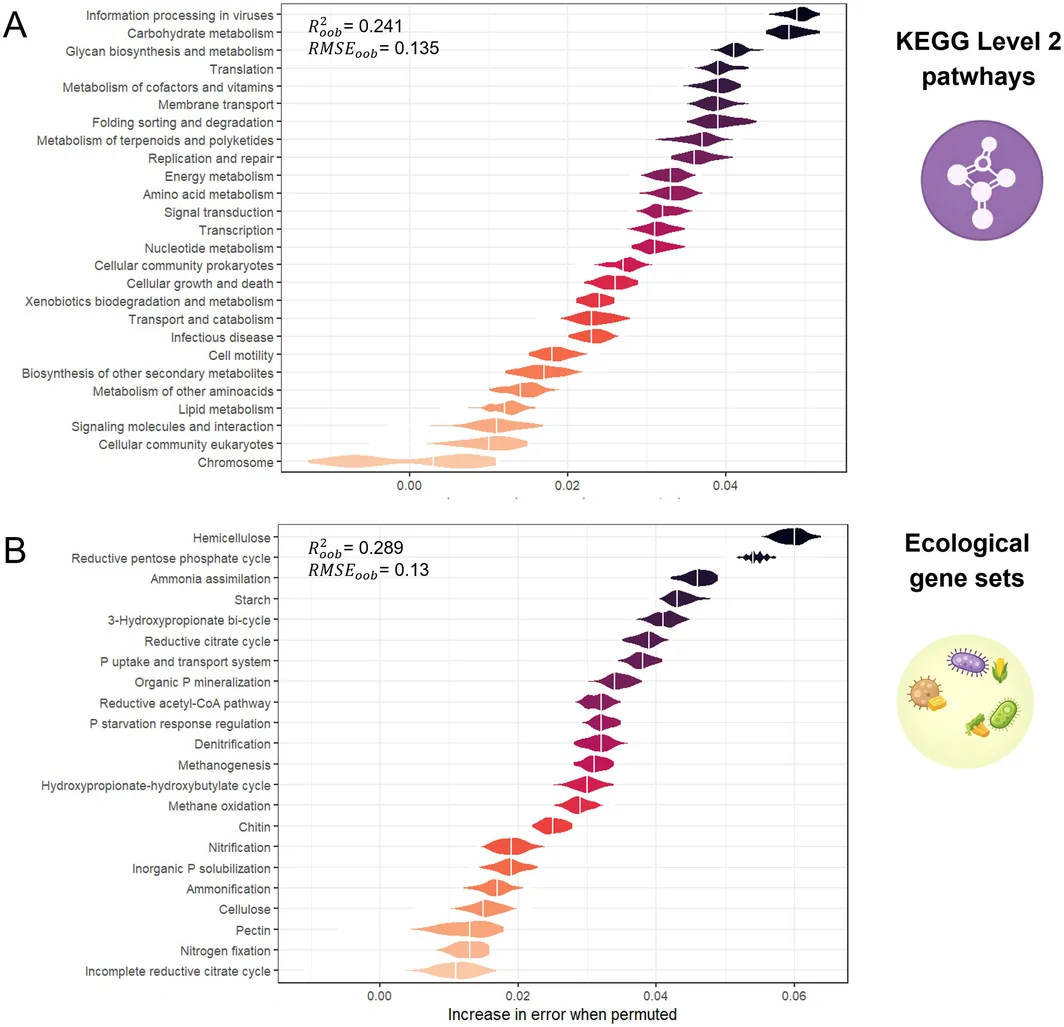
Microbial functional traits as predictors of *Q*_10_ values across global soils. Random Forest analyses showing the relative importance of microbial functional traits in predicting *Q*_10_ values: (A) KEGG Level 2 microbial pathways, and (B) ecologically meaningful gene sets.

**FIGURE 2:**
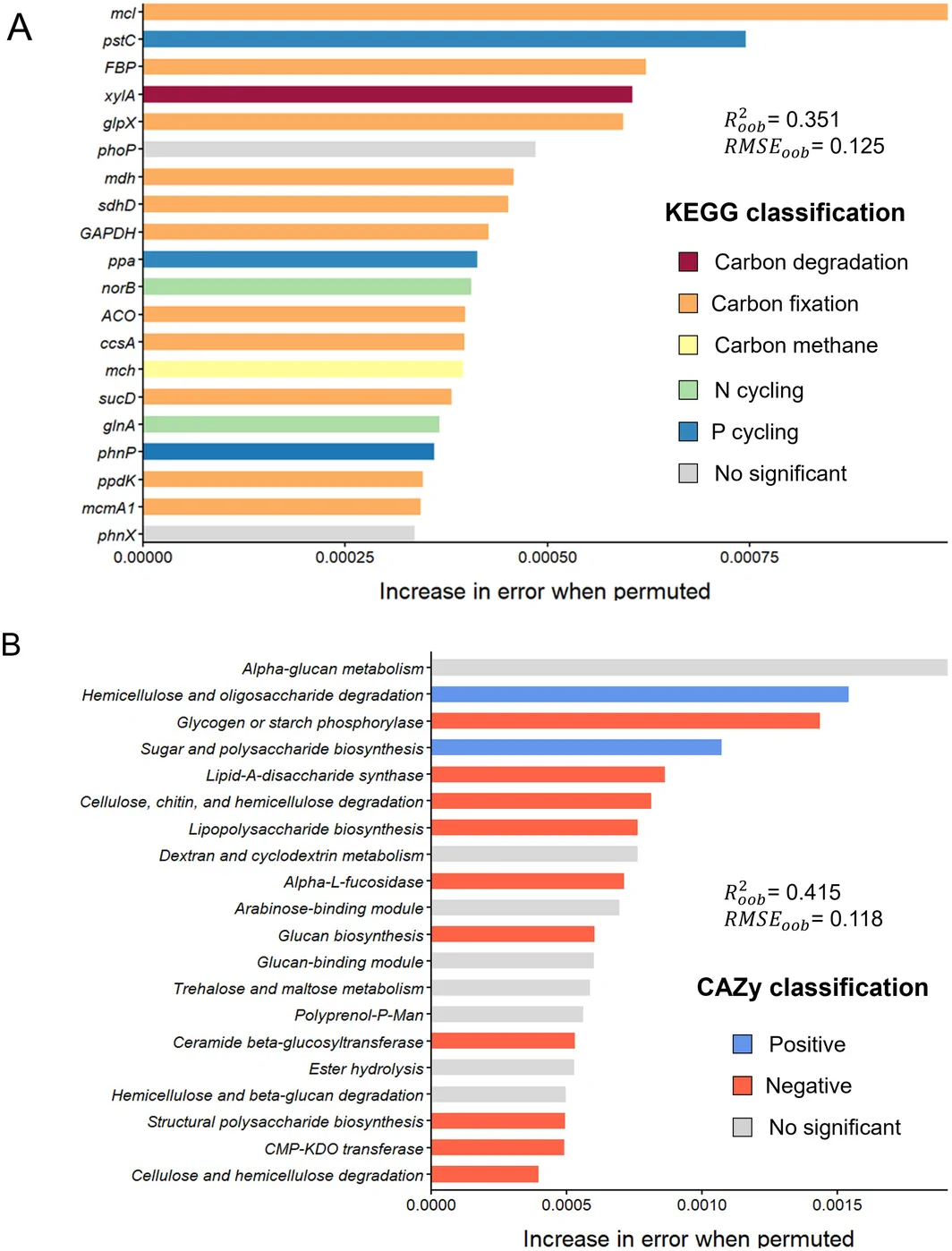
Individual microbial genes as predictors of *Q*_10_ values across global soils. (A) Random Forest analysis showing the top 20 individual genes with the highest relative importance in predicting *Q*_10_ values. Genes are coloured by functional category only for significant genes: Carbon degradation, carbon fixation, carbon–methane, nitrogen cycling, and phosphorus cycling. (B) Top 20 genes associated with *Q*_10_ values based on CAZy classification. Spearman correlations were used to determine negative, positive, and non‐significant associations.

**FIGURE 3:**
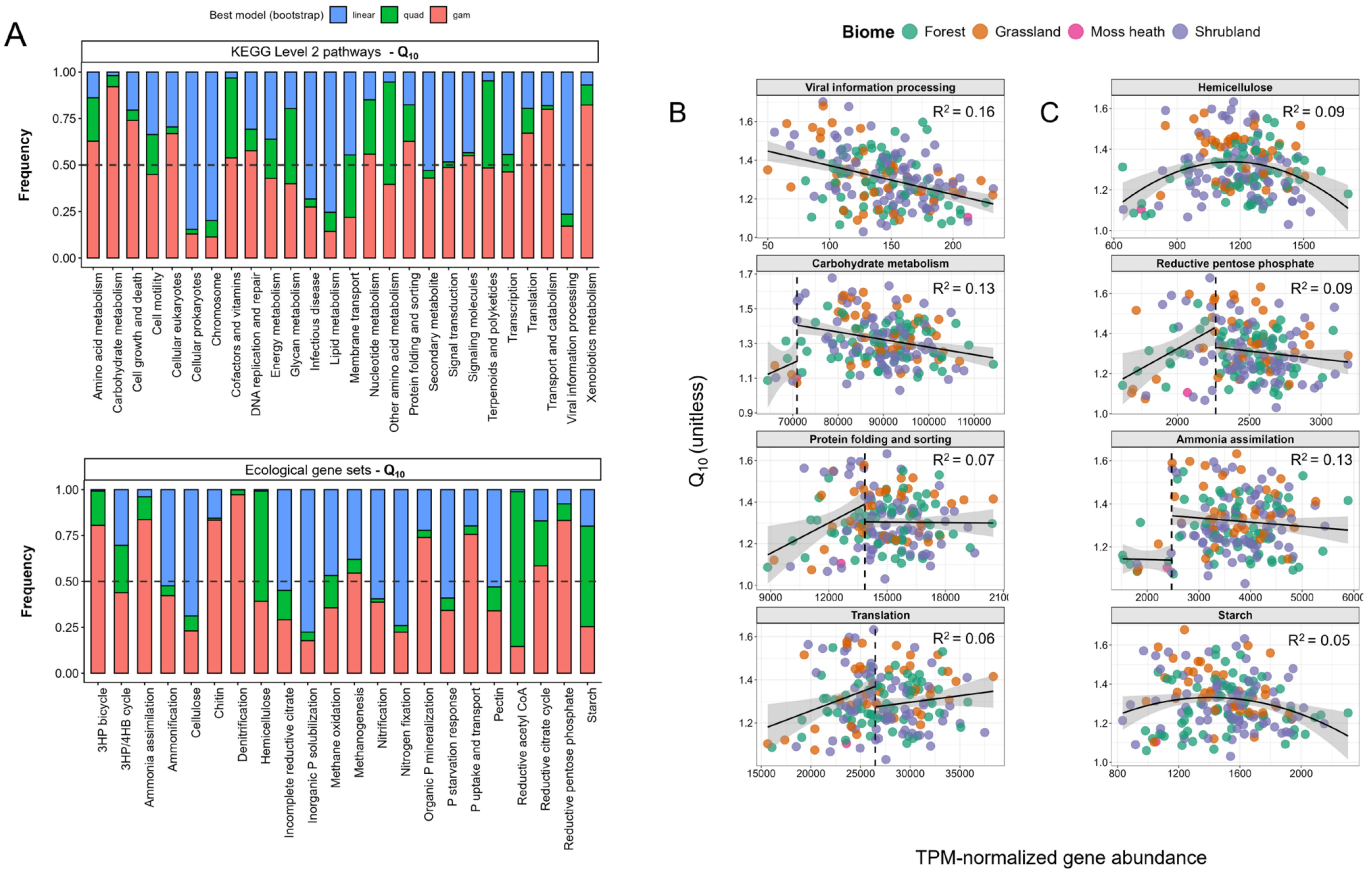
Relationships between microbial functional traits and *Q*_10_ values. (A) Bootstrap‐based model selection for *Q*_10_ values, comparing linear, quadratic, and generalized additive models (GAMs). Frequency on the *y*‐axis indicates how often each model was selected across bootstraps. (B and C) Relationships between *Q*_10_ values (*y*‐axis) and TPM‐normalized abundances (*x*‐axis) of the main predictors from KEGG Level 2 microbial pathways (B) and ecologically meaningful gene sets (C) according to our Random Forest analysis. Vertical lines indicate the functional thresholds identified from non‐linear relationships.

**FIGURE 4:**
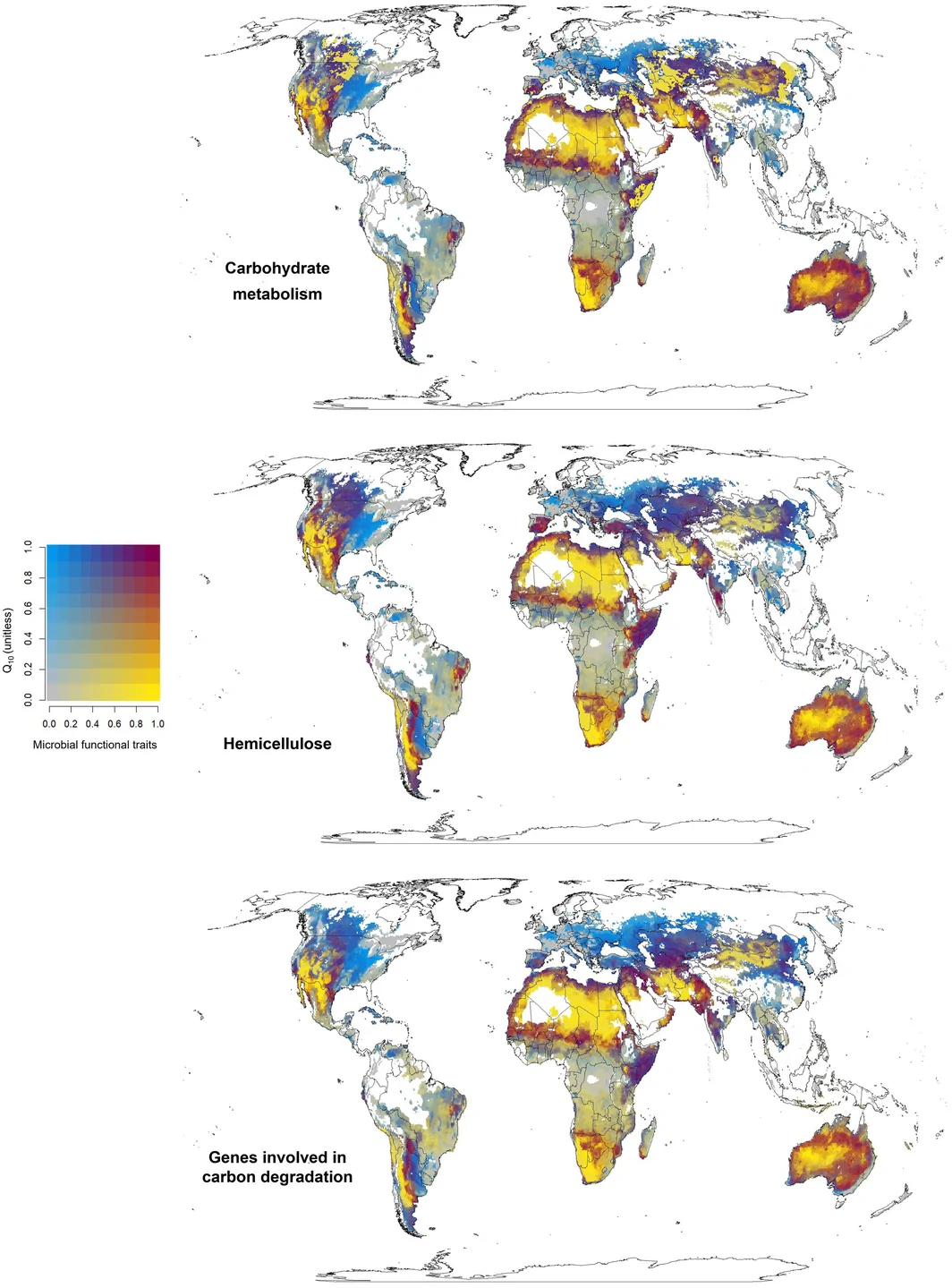
Global distribution of microbial functional traits and *Q*_10_ values. Bivariate maps show the co‐occurrence of *Q*_10_ values (*x*‐axis) and TPM‐normalized abundances of microbial functional traits (*y*‐axis), including carbon metabolism pathways (broad KEGG Level 2; Panel A), hemicellulose degradation (ecologically meaningful gene sets; Panel B), and specific carbon‐degrading genes (Panel C).

By integrating metagenomic data (Chen et al. 2026) and *Q*_10_ values (Sáez‐Sandino et al. 2023), we identified the microbial functional traits most strongly associated with the temperature sensitivity of soil respiration (*Q*_10_). Our analyses reveal that carbohydrate metabolism (KEGG Level 2) and hemicellulose degradation shape *Q*_10_ values across all major biomes (Figures 1 and 2). Moreover, we detected thresholds in the TPM‐normalized abundances of these microbial pathways (Figure 3; individual relationships are shown in Figures S2–S5, and statistical results are provided in Tables S2–S5). This suggests that once these microbial functional traits surpass certain thresholds, even small increases can trigger pronounced positive or negative changes in *Q*_10_ values. Since recent global studies predict shifts in TPM‐normalized abundance of microbial functional traits under future climate scenarios (Chen et al. 2026), our findings have major implications for global carbon cycling. For instance, non‐linear relationships challenge the linear assumptions currently used to link TPM‐normalized abundance of microbial functional traits with soil respiration (He et al. 2020; Liu et al. 2020). Furthermore, the complexity and variability of soil carbon dynamics under warming may be underestimated, as our results suggest that soil carbon loss does not scale proportionally with changes in key microbial functional traits. Finally, our global atlas highlights high‐latitude regions of the Northern Hemisphere (e.g., Canada) where elevated *Q*_10_ values coincide with high TPM‐normalized abundances of microbial genes involved in carbon degradation (Figure 4). Together, high‐latitude regions of the Northern Hemisphere could represent major hotspots of soil carbon loss under future climate scenarios, providing a framework to prioritize regions for monitoring and mitigation.

### 3.1 Main Microbial Functional Traits Shaping *Q*_10_ Values

Broad KEGG classifications revealed that pathways involved in carbohydrate metabolism, particularly those encoding enzymes that degrade carbon‐rich compounds such as polysaccharides, were strongly associated with *Q*_10_ values across global soils (Figure 1A). Consistently, when microbial functions were further classified, the degradation of hemicellulose and starch emerged among the top predictors of *Q*_10_ (Figure 1B). At a finer functional resolution, we found that xylA, encoding xylanase for hemicellulose breakdown, ranked as the fourth most predictive gene for *Q*_10_ values (Figure 2A). Using the Carbohydrate‐Active Enzymes (CAZy) classification, we also found that enzymes linked to labile carbon metabolism such as α‐glucan metabolism, hemicellulose and oligosaccharide degradation, and glycogen or starch phosphorylase showed high predictive power for *Q*_10_ values (Figure 2B). This aligns with ecological theory suggesting that soil microbes preferentially metabolize labile carbon pools to maximize energy yield (Davidson and Janssens 2006). The short‐term nature of the incubations could explain these results, whereby the initial *Q*_10_ values may depend more strongly on microbial functional capacity for labile carbon consumption than on the breakdown of more recalcitrant carbon pools (Domeignoz‐Horta et al. 2023). In fact, enzymes associated with ester hydrolysis, such as carboxylesterases involved in the breakdown of complex xenobiotic compounds and lignin–carbohydrate complexes, showed strong predictive power, yet were negatively associated with *Q*_10_ values. This finding supports the higher energetic cost of degrading recalcitrant compounds. Although these enzymes allow microbes to degrade recalcitrant carbon (Fan et al. 2025), this process could be an energy‐limited microbial strategy, likely employed only when easily degradable substrates are scarce.

Beyond carbon‐degrading metabolism, genes associated with cellular maintenance, such as protein folding, translation and membrane transport, also emerged as strong predictors of *Q*_10_ values (Figure 1A). Other anabolic pathways, including the reductive pentose phosphate cycle and ammonia assimilation, were also strong predictors of *Q*_10_ values (Figure 1B). At finer functional resolution, more than half of the top‐ranking genes were linked to carbon assimilation or energy conservation pathways. For instance, malyl‐CoA lyase (*mcl*), a key enzyme in the glyoxylate cycle that enables the assimilation of C_1_ compounds, was the most important predictor of *Q*_10_ values (Figure 2A). By bypassing CO_2_‐releasing steps of the TCA cycle, the glyoxylate cycle allows microbes to conserve carbon under energy or nutrient limitation (Liao et al. 2023). Similarly, CAZy classification revealed that lipid‐A‐disaccharide synthase was negatively associated with *Q*_10_ values (Figure 2B), likely reflecting a shift of resources towards membrane biosynthesis rather than respiration.

Based on these results, we propose that the identified microbial functional traits converge into three complementary metabolic strategies: (i) carbon acquisition, involving the degradation of labile substrates (e.g., carbohydrate metabolism, hemicellulose degradation and CAZy enzymes); (ii) carbon assimilation, conservation and metabolic efficiency, involving pathways that regulate the allocation and retention of acquired carbon and energy (e.g., the glyoxylate cycle and other carbon assimilation pathways); and (iii) cellular maintenance and stress responses, including processes such as protein folding, membrane transport and translation. Within this framework, microbial communities may allocate carbon and energy among competing metabolic demands. For instance, under carbon‐limited or stressful conditions, microbial responses may involve a metabolic trade‐off: conserving carbon through energy‐efficient pathways (e.g., glyoxylate cycle) or sustaining biosynthesis via anabolic and maintenance processes. These strategies are not mutually exclusive and may operate simultaneously, with their relative balance shaping *Q*_10_ values. Although our observational approach cannot directly test these mechanisms, this framework generates testable hypotheses for future manipulative experiments combining temperature treatments, resource availability and metagenomic analyses.

### 3.2 Thresholds in Microbial Genes Shape *Q*_10_ Values

We then found that the associations between microbial functional traits—across broad KEGG functional categories, ecologically meaningful gene sets, and individual genes—and *Q*_10_ values were consistently non‐linear (Figure 3A). Our non‐linear relationships showed that *Q*_10_ values increased with TPM‐normalized abundance up to a certain threshold, beyond which further increases in TPM‐normalized abundance were associated with declines in *Q*_10_ values (Figure 3B,C). We also found that observations occurring on either side of the thresholds were distributed across multiple ecosystem types (Forest, Grassland, Shrubland and Moss heath; Figure 3B,C), indicating that the identified thresholds were not restricted to a single ecosystem type.

Overall, these non‐linear relationships seem to be consistent with the framework described above. For instance, higher TPM‐normalized abundance of genes involved in hemicellulose and starch degradation may increase the availability of soluble sugars, while ammonia assimilation may support microbial biomass growth (Nelson et al. 2016). However, beyond a functional threshold, further increases in functional gene abundance may be associated with lower *Q*_10_ values as physiological and environmental constraints become increasingly important. These constraints may include osmotic stress, nutrient scarcity (e.g., nitrogen or phosphorus limitation reducing enzyme production), or stoichiometric imbalances (e.g., the predominance of ammonium immobilization over mineralization) (Allison et al. 2010). Under these conditions, microbial communities may increasingly allocate resources towards carbon conservation and cellular maintenance rather than processes directly supporting respiration. Consistent with this interpretation, membrane transport and protein folding showed non‐linear relationships with *Q*_10_ values (Figure 3B), potentially reflecting greater investment in nutrient acquisition and cellular maintenance (e.g., osmoregulation) under stressful conditions. Similarly, the threshold observed in the reductive pentose phosphate pathway suggests a shift towards carbon assimilation and biosynthesis rather than decomposition (Figure 3C). Taken together, our results indicate that low *Q*_10_ values beyond functional thresholds can be explained by the energetic costs of sustaining microbial life, with increased investment in survival and repair functions.

### 3.3 Global Hotspots for Microbial Genes Linked to *Q*_10_ Values

We generated novel global atlases based on the strongest predictors of *Q*_10_ values to identify regions where high *Q*_10_ values co‐occur with elevated TPM‐normalized abundances of (i) carbohydrate metabolism (KEGG Level 2), (ii) specific genes involved in carbon degradation pathways and (iii) hemicellulose breakdown (Figure 4). The highest *Q*_10_ values were located across northern regions of the Northern Hemisphere (e.g., Europe, Siberia and North America), which also represent the largest soil carbon reservoirs on Earth (García‐Palacios et al. 2021). In contrast, arid and hyperarid ecosystems, including the Sahara, western United States and central Australia, displayed high TPM‐normalized abundance of microbial functional traits but low *Q*_10_ values. These global hotspots therefore represent model‐based predictions within the environmental space represented by the training data. Areas primarily located in core hyperarid regions, particularly central areas of the Sahara, and extreme tropical regions were excluded from the final predictions based on Mahalanobis distance (i.e., their environmental conditions fell outside those represented in the training data). Prediction variability among the five cross‐validation models was generally higher for functional gene predictions across tropical ecosystems (Figure S8), whereas variability in *Q*_10_ predictions was highest across northern high‐latitude regions, reaching a maximum of 0.125 (Figure S9). Moreover, our global models further identified distinct hotspots where elevated *Q*_10_ values co‐occur with high TPM‐normalized abundances of carbon‐degrading microbial genes, spanning temperate and continental biomes of northern North America and central Asia, eastern Australia, the Mediterranean basin (encompassing southern Europe and northern Africa) and parts of East Africa (such as Somalia). Regions exhibiting co‐occurrence of high *Q*_10_ values and microbial functional trait abundances showed relatively low prediction variability across cross‐validation models, supporting the consistency of these predicted hotspots.

The positive associations between *Q*_10_ values and microbial functional traits involved in carbon acquisition could provide insights into the global patterns observed. For instance, the strong positive association between hemicellulose and oligosaccharide degradation (e.g., β‐xylosidase) and *Q*_10_ values suggests microbial capacity for xylan degradation across forest soils (López‐Mondéjar et al. 2016). Similarly, the positive association between sugar and polysaccharide biosynthesis and *Q*_10_ values may reflect the role of root‐derived sucrose in supporting microbial respiration in rhizosphere soils across increasing temperatures. Together, these associations suggest that microbial capacity for carbon acquisition and processing may contribute to the high *Q*_10_ values observed in carbon‐rich ecosystems. In contrast, the apparent decoupling between *Q*_10_ values and microbial functional traits in arid regions may reflect microbial adaptation to water limitation and low substrate availability. Microbial communities in these environments may maintain genes involved in carbon assimilation to support basal metabolism while retaining the capacity for opportunistic carbon use during brief and sporadic rainfall events (Vásquez‐Dean et al. 2020). In tropical ecosystems, particularly in the Amazon and Congo Basin, we observed intermediate values of both TPM‐normalized gene abundance and *Q*_10_ values. This pattern may indicate that microbial communities in these regions operate under relatively balanced resource conditions, where neither carbon limitation nor substrate excess strongly dominates microbial activity.

### 3.4 New Insights Into the Role of Soil Viral Dynamics in Shaping *Q*_10_ Values

Using qPCR data available for a subset of 173 samples (Xiong et al. 2026), Random Forest analyses further identified bacterial abundance (16S rRNA copy number) as the second strongest predictor of *Q*_10_ values after viral information‐processing genes (KEGG Level 2) (Figure S6). First, we found a negative linear relationship between *Q*_10_ values and the abundance of viral information‐processing genes (Figure 3B). One potential mechanism is through interactions between viruses and their microbial hosts, which could influence microbial biomass and community structure. Second, the relationship between *Q*_10_ values and bacterial abundance was non‐linear. We found that *Q*_10_ values declined with increasing bacterial abundance up to a threshold of 9.53 (log_10_ copies g^−1^ soil), beyond which they increased linearly with further increases in bacterial abundance (Figure S6). Third, the abundance of viral information‐processing genes showed a negative relationship with bacterial abundance (16S rRNA copy number) (Figure S7). One possible explanation for these patterns is that viral–microbial interactions may influence *Q*_10_ values through changes in microbial biomass. Under conditions of lower viral pressure, microbial populations may be able to maintain greater biomass, potentially supporting stronger temperature‐sensitive respiratory responses (Osburn et al. 2024). Conversely, stronger viral pressure could reduce microbial biomass through host‐cell lysis, potentially decreasing the proportion of metabolically active cells and constraining *Q*_10_ values (Albright et al. 2022).

Overall, these findings suggest a potential pathway whereby viral–microbial interactions could indirectly influence *Q*_10_ values through their effects on microbial biomass or community structure. However, metagenomic data provides information on the relative abundance of genes associated with viral functional processes rather than direct measurements of viral abundance or activity. Moreover, our observational data reveal associations among viral functional traits, bacterial abundance, and *Q*_10_ values, but do not demonstrate a causal role of viral processes in regulating microbial respiration. Experiments manipulating viral abundance or activity, including community coalescence of soils with contrasting viral functional gene abundance, could directly test the potential role of viral–microbial interactions in shaping *Q*_10_ values. Future studies combining these experimental approaches with viromics, direct measurements of viral abundance and activity, viral lysis rates, microbial biomass and respiration will be needed to determine whether viral–microbial interactions play a significant yet underappreciated role in soil carbon–climate feedbacks.

### 3.5 Microbial Methane Cycling Associated With the MAOC : POC Ratio

For a subset of 179 soil samples (Sáez‐Sandino, Gallardo, et al. 2024; Sáez‐Sandino, Maestre, et al. 2024), we also assessed the association between TPM‐normalized abundance of microbial functional traits and the MAOC : POC ratio (Figure S8). This ratio provides an indicator of soil carbon stabilization by capturing the balance between mineral‐associated organic carbon, which is relatively protected from microbial decomposition, and particulate organic carbon, which is generally more accessible to microbial mineralization (Lavallee et al. 2020). Our Random Forest analyses identified methane oxidation and methanogenesis as the most important predictors of the MAOC : POC ratio (Figure S9). Moreover, we also found that both microbial pathways were positively and linearly associated with the MAOC : POC ratio. These associations suggest that methane‐cycling microbial functional traits may be linked to patterns of soil carbon stabilization. One possible explanation is that methane‐cycling microorganisms contribute to carbon retention through microbial biomass and necromass formation, which can ultimately contribute to mineral‐associated organic carbon (Gentsch et al. 2018). An alternative explanation is that shared environmental conditions (soil texture, moisture status and/or anoxic conditions) may influence both methane‐cycling microbial communities and stabilization or preservation of organic carbon. For instance, the strong association of methanogenesis with anoxic conditions suggests that the relationship between methane‐cycling traits and the MAOC ratio may be especially relevant in waterlogged and oxygen‐limited environments (Angle et al. 2017), including some high‐latitude and permafrost soils. Future studies targeting permafrost and other high‐latitude ecosystems, and combining metagenomic analyses with methane flux measurements, isotopic tracing, and direct measurements of microbial carbon assimilation and necromass formation, will be needed to determine whether methane‐cycling microorganisms contribute directly to the stabilization of organic carbon and how these processes may influence carbon vulnerability under climate change.

### 3.6 Limitations and Future Directions

As with any global ecological study, our analysis is not without limitations. First, our *Q*_10_ values were obtained under standardized laboratory conditions and should therefore not be interpreted as direct estimates of field responses to climate warming. The 10‐h incubation period was selected to minimize changes in substrate availability and microbial physiological state that can emerge during prolonged incubations, while facilitating comparisons among soils originating from contrasting biomes. However, short‐term incubations cannot capture longer term processes that may influence *Q*_10_ over time, including microbial acclimation, shifts in community composition, changes in substrate availability and interactions with soil moisture and mineralogy (Niu et al. 2024; Sáez‐Sandino et al. 2025; Zhou et al. 2014). We additionally performed a quantitative comparison of our *Q*_10_ values with the global meta‐analysis of Haaf et al. (2021), which includes measurements obtained across different durations. The *Q*_10_ values in our study were generally lower than those reported for longer duration experiments, with median values ranging from 1.1 to 1.6 (Figure S12). This comparison suggests that our standardized short‐term measurements do not necessarily produce inflated *Q*_10_ estimates, while also highlighting that *Q*_10_ values can vary substantially with experimental duration and context. Future studies combining long‐term warming experiments with metagenomic analyses will be essential to determine how microbial functional traits contribute to the temperature sensitivity of soil respiration under prolonged warming.

In summary, by combining temperature‐gradient incubations with metagenomic analyses across all major biomes, our study provides a identifies microbial functional traits associated with *Q*_10_ values. Our results suggest that microbial functional traits can be grouped into three complementary metabolic strategies: carbon acquisition, carbon assimilation and conservation, and cellular maintenance and stress responses. Carbohydrate metabolism and hemicellulose degradation emerged as the strongest predictors of global *Q*_10_ patterns, highlighting the potential importance of microbial carbon acquisition. Under carbon‐limited or stressful conditions, microbial communities may increasingly allocate resources towards carbon conservation, cellular maintenance (e.g., protein folding and membrane biosynthesis) and stress‐tolerance pathways (e.g., glycan biosynthesis and activation of carboxylesterases for recalcitrant carbon degradation). The balance among these complementary strategies may contribute to variation in *Q*_10_ values across environmental conditions. Consistent with this framework, *Q*_10_ values showed non‐linear relationships with microbial functional traits, with abrupt shifts occurring when specific thresholds in TPM‐normalized gene abundance were crossed. These thresholds were observed across multiple ecosystem types, suggesting that the identified non‐linear patterns were not restricted to a single biome. Our global mapping further revealed that regions with high *Q*_10_ values and elevated abundances of carbon‐degrading microbial genes are concentrated in high‐latitude regions of the Northern Hemisphere, which contain some of the largest soil carbon reservoirs globally. These patterns represent model‐based predictions of regions where microbial functional traits and *Q*_10_ values co‐occur, which may indicate potential vulnerability of carbon‐rich ecosystems to future temperature increases. For a subset of soils, methane oxidation and methanogenesis were also strongly associated with the MAOC : POC ratio, suggesting a potential link between methane‐cycling microbial traits and patterns of soil carbon stabilization. Whether methane‐cycling microorganisms directly contribute to carbon stabilization remains an open question requiring targeted experimental validation, particularly in high‐latitude and permafrost ecosystems. Finally, viral information‐processing genes and bacterial abundance were among the strongest predictors of *Q*_10_ values. These associations raise the possibility that viral–microbial interactions could indirectly influence *Q*_10_ values through their effects on microbial biomass. Overall, our findings provide a foundation for incorporating microbial functional traits into models of global soil carbon dynamics and highlight testable hypotheses linking microbial metabolism, methane cycling and viral–microbial interactions to the temperature sensitivity of soil respiration.

## Author Contributions

**Tadeo Sáez‐Sandino:** conceptualization, investigation, writing – review and editing, writing – original draft, data curation, methodology, formal analysis, visualization. **Brajesh K. Singh:** funding acquisition, writing – review and editing, writing – original draft, investigation, conceptualization. **Chao Xiong:** writing – original draft, writing – review and editing, software, data curation, methodology. **Juntao Wang:** writing – original draft, writing – review and editing, software, data curation, methodology. **Chengjie Ren:** writing – original draft, writing – review and editing. **Manuel Delgado‐Baquerizo:** writing – original draft, writing – review and editing, funding acquisition, project administration, conceptualization, investigation.

## Funding

B.K.S. and T.S.‐S. are supported by Australian Research Council (DP230101448), which funded metagenomics work. T.S.‐S., and M.D.‐B. are supported by TED2021‐130908B‐C41 (URBANCHANGE) and a project PAIDI 2020 from the Junta de Andalucía (P20_00879). M.D.‐B. was also supported by the European Research Council (ERC) grant 647038 (BIODESERT), the BES grant agreement No LRB17\1019 (MUSGONET), innovation programme under Marie Sklodowska‐Curie Grant Agreement 702057 (CLIMIFUN), and a project from the Spanish Ministry of Science and Innovation (PID2020‐115813RA‐I00; SOIL4GROWTH). C.X. was supported by ARC Discovery Early Career Researcher Award (DE260100677).

## Conflicts of Interest

The authors declare no conflicts of interest.

## Supporting information


**Figure S1:** Global distribution of soil samples (*n* = 196). Points represent soil samples collected across diverse terrestrial ecosystems worldwide for the field survey presented in this study.
**Figure S2:** Bootstrap‐based model selection of *Q*_10_ based on KEGG Level 2 pathways. Metagenomic data are represented by TPM‐normalized abundances of KEGG Level 2 pathways. The *y*‐axis shows the frequency with which each model was selected across bootstrap resamples, indicating the consistency of model selection among bootstrap iterations.
**Figure S3:** Relationships between *Q*_10_ values and KEGG Level 2 pathways. Vertical lines indicate the functional thresholds identified from non‐linear relationships. Statistical results are provided in Table S2.
**Figure S4:** Bootstrap‐based model selection of *Q*_10_ based on ecologically meaningful gene sets. Metagenomic data are represented by TPM‐normalized abundances of KEGG Level 2 pathways. The *y*‐axis shows the frequency with which each model was selected across bootstrap resamples, indicating the consistency of model selection among bootstrap iterations.
**Figure S5:** Relationships between *Q*_10_ values and ecologically meaningful gene sets. Vertical lines indicate the functional thresholds identified from non‐linear relationships. Statistical results are provided in Table S3.
**Figure S6:** Microbial functional pathways and 16S rRNA gene abundance as predictors of *Q*_10_ values (*n* = 173). (A) Frequency of model selection across bootstrap resamples, indicating the consistency of model selection. (B) Random Forest analysis identifies the importance of individual predictors for *Q*_10_ values. (C) Relationship between *Q*_10_ values and 16S rRNA gene abundance measured by quantitative PCR (qPCR). Statistical results are provided in Table S4.
**Figure S7:** 16S rRNA gene abundance as a predictor of viral information‐processing pathways (*n* = 173). (A) Frequency of model selection across bootstrap resamples, indicating the consistency of the selected model. (B) Relationship between 16S rRNA gene abundance and TPM‐normalized abundance of viral information‐processing pathways. Statistical results are provided in Table S4.
**Figure S8:** Bootstrap‐based model selection of MAOC : POC ratio based on ecologically meaningful gene sets. The *y*‐axis shows the frequency with which each model was selected across bootstrap resamples, indicating the consistency of model selection among bootstrap iterations.
**Figure S9:** Microbial functional pathways as predictors of MAOC : POC ratio (*n* = 179). (A) Random Forest analysis identifies the importance of individual predictors. (B) Linear relationships between TPM‐normalized abundances of methane oxidation and methanogenesis genes and the MAOC : POC ratio. Statistical results are provided in Table S5.
**Figure S10:** Uncertainty maps of microbial functional traits. (A) Predicted global distribution of microbial functional traits at a 25 km spatial resolution, based on machine learning models using data from 196 sampling sites.
**Figure S11:** Uncertainty maps of *Q*_10_ values. (A) Predicted global distribution of microbial functional traits at a 25 km spatial resolution, based on machine learning models using data from 196 sampling sites.
**Figure S12:** Comparison of *Q*_10_ values across incubation durations and experimental conditions. Boxplots show the distribution of heterotrophic soil respiration *Q*_10_ values reported in the global meta‐analysis of Haaf et al. (2021) and our study. Results were grouped by incubation duration (short‐term, ≤ 1 day; medium‐term, 1–30 days; long‐term, > 30 days) and experimental approach (laboratory incubations versus field warming experiments). Centre lines indicate medians and box limits indicate the 25th and 75th percentiles.
**Table S1:** List of microbial functional traits used in this study.
**Table S2:** Statistical results for the relationships between *Q*_10_ values and KEGG Level 2 pathways. The initial model was selected from linear, quadratic (quad) or generalized additive models (GAMs). The table reports the coefficient of determination (*R* ^2^) and *p* values for each model.
**Table S3:** Statistical results for the relationships between *Q*_10_ values ecologically meaningful gene sets. The initial model was selected from linear, quadratic (quad) or generalized additive models (GAMs). The table reports the coefficient of determination (*R* ^2^) and *p* values for each model.
**Table S4:** Statistical results for 16S rRNA gene abundance and of viral information‐processing pathways. The initial model was selected from linear, quadratic (quad) or generalized additive models (GAMs). The table reports the coefficient of determination (*R* ^2^) and *p* values for each model.
**Table S5:** Statistical results for the MAOC : POC ratio. The initial model was selected from linear, quadratic (quad) or generalized additive models (GAMs). The table reports the coefficient of determination (*R* ^2^) and *p* values for each model.
**Table S6:** Performance metrics and spatial autocorrelation diagnostics for Random Forest models. Model performance is reported as the coefficient of determination between observed and predicted values (*R* ^2^_obs–pred), variance explained (Var_%), holdout validation statistics (*R* ^2^, RMSE and MAE), and Moran's *I* values calculated for both the response variables and out‐of‐bag (OOB) residuals using *k*‐nearest neighbour spatial weights.

## Data Availability

A detailed list of all KEGG entries, including their hierarchical level (e.g., Level 2 pathways) and the selection of individual genes representing ecologically meaningful processes is provided this database (https://figshare.com/s/daaf9aacc40650089fd6). All raw sequencing data used in this study have been submitted to the NCBI Sequence Read Archive (SRA) database under the accession numbers PRJNA1162941 (shotgun metagenomics).
