# Supplementary material for: Microbial Functional Genes Help Explain Soil Carbon Sensitivity to Temperature Across Global Biomes
Source: Glob Chang Biol. 2026 Sep 23;32(9):e71101. doi: 10.1111/gcb.71101 (PMC13601813; doi:10.1111/gcb.71101)
Supplement: Supplementary file 1 [file GCB-32-e71101-s001.docx]

**This PDF file includes:**

Supplementary Figures S1 to S12

Supplementary Table S1 to S6

**Figure S1 | Global distribution of soil samples (n =196).** Points represent soil samples collected across diverse terrestrial ecosystems worldwide for the field survey presented in this study.

**Figure S2 | Bootstrap-based model selection of Q₁₀ based on KEGG level 2 pathways.** Metagenomic data is represented by TPM-normalized abundances of KEGG level 2 pathways. The y-axis shows the frequency with which each model was selected across bootstrap resamples, indicating the consistency of model selection among bootstrap iterations.

**Figure S3 | Relationships between Q₁₀ values and KEGG level 2 pathways.** Vertical lines indicate the functional thresholds identified from nonlinear relationships. Statistical results are provided in Table S2.

**Figure S4 | Bootstrap-based model selection of Q₁₀ based on ecologically meaningful gene sets.** Metagenomic data is represented by TPM-normalized abundances of KEGG level 2 pathways. The y-axis shows the frequency with which each model was selected across bootstrap resamples, indicating the consistency of model selection among bootstrap iterations.

**Figure S5 | Relationships between Q₁₀ values and ecologically meaningful gene sets.** Vertical lines indicate the functional thresholds identified from nonlinear relationships. Statistical results are provided in Table S3.

**Figure S6 | Microbial functional pathways and 16S rRNA gene abundance as predictors of Q₁₀ values (n= 173)**. A) Frequency of model selection across bootstrap resamples, indicating the consistency of model selection. B) Random Forest analysis identifies the importance of individual predictors for Q₁₀ values. C) Relationship between Q₁₀ values and 16S rRNA gene abundance measured by quantitative PCR (qPCR). Statistical results are provided in Table S4.

**Figure S7 | 16S rRNA gene abundance as a predictor of viral information-processing pathways (n= 173).** A) Frequency of model selection across bootstrap resamples, indicating the consistency of the selected model. B) Relationship between 16S rRNA gene abundance and TPM-normalized abundance of viral information-processing pathways. Statistical results are provided in Table S4.

**Figure S8 | Bootstrap-based model selection of MAOC: POC ratio based on ecologically meaningful gene sets.** The y-axis shows the frequency with which each model was selected across bootstrap resamples, indicating the consistency of model selection among bootstrap iterations.

**Figure S9 | Microbial functional pathways as predictors of MAOC: POC ratio (n = 179).** (A) Random Forest analysis identifies the importance of individual predictors. (B) Linear relationships between TPM-normalized abundances of methane oxidation and methanogenesis genes and the MAOC:POC ratio. Statistical results are provided in Table S5.

**Figure S10 |** **Uncertainty maps of microbial functional traits.** (A) Predicted global distribution of microbial functional traits at a 25 km spatial resolution, based on machine learning models using data from 196 sampling sites.

**Figure S11 |** **Uncertainty maps of Q_10_ values.** (A) Predicted global distribution of microbial functional traits at a 25 km spatial resolution, based on machine learning models using data from 196 sampling sites.

**Figure S12 | Comparison of Q₁₀ values across incubation durations and experimental conditions**. Boxplots show the distribution of heterotrophic soil respiration Q₁₀ values reported in the global meta-analysis of Haaf et al. (2021) and our study. Results were grouped by incubation duration (short-term, ≤1 day; medium-term, 1–30 days; long-term, >30 days) and experimental approach (laboratory incubations versus field warming experiments). Centre lines indicate medians and box limits indicate the 25th and 75th percentiles.

**Table S1. List of microbial functional traits used in this study.** Supplementary Table S1 is available online as a Separate .XLS file under the Supplementary Information for this article.

**Table S2 | Statistical results for the relationships between Q₁₀ values and KEGG level 2 pathways.** The initial model was selected from linear, quadratic (quad) or generalized additive models (GAMs). The table reports the coefficient of determination (R²) and P values for each model.

**Table S3 | Statistical results for the relationships between Q₁₀ values ecologically meaningful gene sets..** The initial model was selected from linear, quadratic (quad) or generalized additive models (GAMs). The table reports the coefficient of determination (R²) and P values for each model.

**Table S4 | Statistical results for 16S rRNA gene abundance and of viral information-processing pathways.** The initial model was selected from linear, quadratic (quad) or generalized additive models (GAMs). The table reports the coefficient of determination (R²) and P values for each model.

**Table S5 | Statistical results for the MAOC: POC ratio.** The initial model was selected from linear, quadratic (quad) or generalized additive models (GAMs). The table reports the coefficient of determination (R²) and P values for each model.

**Table S6 | Performance metrics and spatial autocorrelation diagnostics for Random Forest models**. Model performance is reported as the coefficient of determination between observed and predicted values (R²_obs–pred), variance explained (Var_%), holdout validation statistics (R², RMSE and MAE), and Moran’s I values calculated for both the response variables and out-of-bag (OOB) residuals using k-nearest neighbour spatial weights.
